# The Significance of *MGMT* Promoter Methylation Status in Diffuse Glioma

**DOI:** 10.3390/ijms232113034

**Published:** 2022-10-27

**Authors:** Nikola Jovanović, Milica Lazarević, Vladimir J. Cvetković, Vesna Nikolov, Jelena Kostić Perić, Milena Ugrin, Sonja Pavlović, Tatjana Mitrović

**Affiliations:** 1 Laboratory for Molecular Biology and Biotechnology, Department of Biology and Ecology, Faculty of Sciences and Mathematics, University of Niš, 18000 Niš, Serbia; 2 Faculty of Medicine, Clinic of Neurosurgery, Clinical Center, University of Niš, 18000 Niš, Serbia; 3 Laboratory for Molecular Biomedicine, Institute of Molecular Genetics and Genetic Engineering, University of Belgrade, 11042 Belgrade, Serbia

**Keywords:** diffuse glioma, glioblastoma, *MGMT*, *IDH1*, *IDH2*, MSP, qMSP

## Abstract

A single-institution observational study with 43 newly diagnosed diffuse gliomas defined the isocitrate dehydrogenase 1 and 2 (*IDH1/2*) gene mutation status and evaluated the prognostic relevance of the methylation status of the epigenetic marker O^6^-methylguanine-DNA methyltransferase (*MGMT*). Younger patients (<50 years) with surgically resected glioma and temozolomide (TMZ) adjuvant chemotherapy were associated with better prognosis, consistent with other studies. The methylation status depends on the chosen method and the cut-off value determination. Methylation-specific PCR (MSP) established the methylation status for 36 glioma patients (19 (52.8%) positively methylated and 17 (47.2%) unmethylated) without relevancy for the overall survival (OS) (*p* = 0.33). On the other side, real-time methylation-specific PCR (qMSP) revealed 23 tumor samples (54%) that were positively methylated without association with OS (*p* = 0.15). A combined MSP analysis, which included the homogenous cohort of 24 patients (>50 years with surgical resection and *IDH1/2*-wildtype diffuse glioma), distinguished 10 (41.6%) methylated samples from 14 (58.4%) unmethylated samples. Finally, significant correlation between OS and methylation status was noticed (*p* ≈ 0.05). The OS of the hypermethylated group was 9.6 ± 1.77 months, whereas the OS of the unmethylated group was 5.43 ± 1.04 months. Our study recognized the *MGMT* promoter methylation status as a positive prognostic factor within the described homogenous cohort, although further verification in a larger population of diffuse gliomas is required.

## 1. Introduction

Diffuse gliomas are highly invasive infiltrating tumors of the central nervous system (CNS) that originate from glial cells. Without a clear boundary between the tumor and the surrounding tissues, diffuse gliomas cannot be completely surgically resected and have a tendency to recur. Based on histological and molecular distinctions, the latest World Health Organization (WHO) classification of CNS tumors (5th edition, 2021) recognizes adult- and pediatric-type diffuse gliomas [1]. Among adult-type diffuse gliomas, glioblastoma (GBM, WHO grade 4) is considered as the most common (54% of all malignant tumors of the CNS), the most aggressive, and the most fatal malignant glioma (the median life expectancy is 8 months) [2,3]. Traditionally, GBMs are further classified into primary and secondary GBMs. The vast majority of GBMs (90–95%) belong to primary GBMs and rapidly arise de novo without recognizable precursor lesions, predominantly in elderly patients with a short survival time [4]. Secondary GBMs (5–10% of GBMs) evolve from lower-grade gliomas (LGGs; diffuse astrocytoma (WHO grade 2) and anaplastic astrocytoma (WHO grade 3) over 5 years in younger patients and have a better prognosis [4]. Although histologically indistinguishable, primary and secondary GBMs are genetically and epigenetically different [4]. Thus, the latest WHO classification of diffuse gliomas recognized the need to combine histological and molecular diagnoses into integrated and layered diagnoses [1]. Common adult-type diffuse gliomas are divided into three types: astrocytoma, isocitrate dehydrogenase (*IDH*)-mutant; oligodendroglioma, *IDH*-mutant and 1p/19q-codeleted; and glioblastoma, *IDH*-wildtype [1].

The above-mentioned *IDH* mutation has been considered to be the most clinically relevant glioma marker since its observation by Parson et al. in 2008 and its recognition as the earliest genetic event driver in gliomagenesis [5,6]. *IDH* mutations are hallmarks of secondary GBMs (78%) and LGGs (diffuse astrocytoma (75%) and anaplastic astrocytoma (85%)) and are rarely determined in primary GBMs (3%) [3]. Furthermore, in The Cancer Genome Atlas (TCGA), which first introduced the molecular classification of GBMs based on gene expression as proneural, neural, classical, and mesenchymal, the *IDH* mutation is considered to be a proneural marker and a sign of good prognosis [7]. Notably, *IDH* mutations are associated with a better prognosis throughout every grade of glioma [8]. Moreover, *IDH* mutations are detected in many other malignancies, such as chondrosarcoma (50%), acute myeloid leukemia (AML; 30%), cholangiocarcinoma (20%), angioimmunoblastic T-cell lymphoma (AITL), solid papillary carcinoma with reverse polarity (SPCRP), melanoma, breast cancer, etc. [9].

The *IDH* gene family encodes three isomerases: *IDH1*, *IDH2,* and *IDH3*. The first, nicotinamide adenine dinucleotide phosphate (NADP) (+)-dependent isocitrate dehydrogenase 1 (*IDH1*) is located in the cytosol and peroxisome and is predominantly expressed in the liver [9]. The other two (*IDH 2* and *IDH3*) are restricted to the mitochondria and are expressed in heart and muscle tissues and lymphocytes [9]. Wildtype *IDH* catalyzes the NADP(+)-dependent oxidative decarboxylation of isocitrate to α-ketoglutarate (α-KG), producing CO_2_ and NADPH within the Krebs cycle [9]. Hence, normal *IDH* is a major producer of NADPH, which is essential for the regeneration of reduced glutathione, known as the main antioxidant in the cell [9]. On the other side, mutated IDH increases oxidative stress and DNA damage [9].

The hotspots for *IDH* mutations are the Arg residues responsible for substrate recognition (R132 for *IDH1*; R140 and R172 for *IDH2*) [9]. The most frequent mutation is a heterozygous point mutation of *IDH1-IDH1*R132H (90% of cases), which affects codon 132 of the gene, located on chromosome 2q33, resulting in an Arg-to-His substitution and decreased affinity for isocitrate [8]. A mutated *IDH1/2* gain of function catalyzes the conversion of α-KG into D-2-hydroxyglutarate (2-HG). An increased concentration of the oncometabolite 2-HG competitively inhibits the activity of α-KG-dependent dioxygenases, such as JmjC (JumonjiC) domain-containing histone demethylases (KDMs) and the TET (ten-eleven translocation) family of DNA hydroxylases, leading to increased histone H3 lysine methylation and cytosine–guanine dinucleotide (CpG) methylation (i.e., hypermethylator phenotype of ~20% of glioma CpG islands or G-CIMP) and an ultimate malignant transformation [10].

Briefly, an initiating genetic mutation, such as the IDH1/2 mutation, is an overture to the complex and dynamic interplay between metabolic, epigenetic, and genetic alterations, leading to a global dysregulation of gene expression (linked to cell cycle regulation, DNA repair mechanisms, apoptosis, cell differentiation, metabolic pathways, and redox homeostasis) and consequently gliomagenesis and metastasis with characteristic malignant GBM heterogeneity, plasticity, vascularity, invasiveness, and aggressiveness [10,11,12,13].

One of the first and thus best-studied epigenetic modifications in the wide spectrum of neoplasia, including gliomas, is O^6^-methylguanine-DNA methyltransferase (*MGMT*) gene promoter methylation [14,15]. It is estimated that the human genome contains 3 × 10^7^ CpG doublets, mostly clustered in CpG islands (CGI) that are susceptible to DNA methylation [16]. The *MGMT* promoter contains 97 CpG sites within a 777 bp-long CGI [17,18]. The aberrant methylation of the *MGMT* promoter in 40% of gliomas leads to the transcriptional repression of the gene and consequently to the loss of the encoded DNA repair enzyme that removes alkylating lesions induced by chemotherapeutic alkylating agents (such as bis-chloroethylnitrosourea (BCNU) and temozolomide (TMZ)) [15]. The fact that more than 90 % of *IDH*-mutant gliomas exhibit a hypermethylated *MGMT* profile is an excellent indication that in these cases the *MGMT* epigenetic alteration is a consequence of *IDH* mutations and 2-HG accumulation [10,19]. Significantly, the hypermethylation of the *MGMT* promoter and low *MGMT* expression may play a crucial role in glioma occurrence and progression and were found to be responsible for the favorable prognosis of LGGs and GBMs treated with TMZ with or without additional radiotherapy [19,20].

Over 50% of GBM patients treated with TMZ do not respond to the therapy. Such resistance is driven primarily by a relatively small population of highly tumorigenic cancer stem cells—glioma stem cells (GSCs) [21]. Although the main molecular factor causing the TMZ resistance of GSCs seems to be MGMT activity, data suggest that other factors could complement its activity through various molecular mechanisms. For example, several genes that encode mismatch DNA repair mechanism (MMR) proteins (MSH2, MSH6, PMS2, and MLH1) could drive TMZ resistance by acquiring mutations both de novo and in response to standard chemotherapeutic treatment [21]. Considering this, our research group recognizes the importance of mismatch repair deficiency analyses in determining the prognostic significance of *MGMT* promoter methylation status. Thus, it will be included in our future studies, further exploring the *MGMT* methylation prognostic significance.

The aim of this study was to make a new effort to resolve the dilemma of whether the *MGMT* promoter methylation status has prognostic value for diffuse glioma samples from Serbian patients. Previously, although a significant improvement was observed in overall survival with TMZ treatment (the median survival was 15 months) in comparison with diffuse glioma patients without treatment (the median survival was 5 months), it was not interrelated with the *MGMT* promoter methylation status [22,23]. Currently, the *MGMT* promoter methylation status is investigated in the context of the clinical data, the choice of method for its assessment (real-time methylation-specific PCR (qMSP) vs. previous methylation-specific PCR (MSP)), and the *IDH1*/2 mutation status.

## 2. Results

### 2.1. Clinical Characteristics

This study is a single-institution observational study that involved 45 brain tumor samples from newly diagnosed and treated patients at the Neurosurgery Clinic of the University Clinical Centre of Niš before the COVID-19 pandemic. The list of brain tumor samples is given in Table 1. 

**Figure 1:**
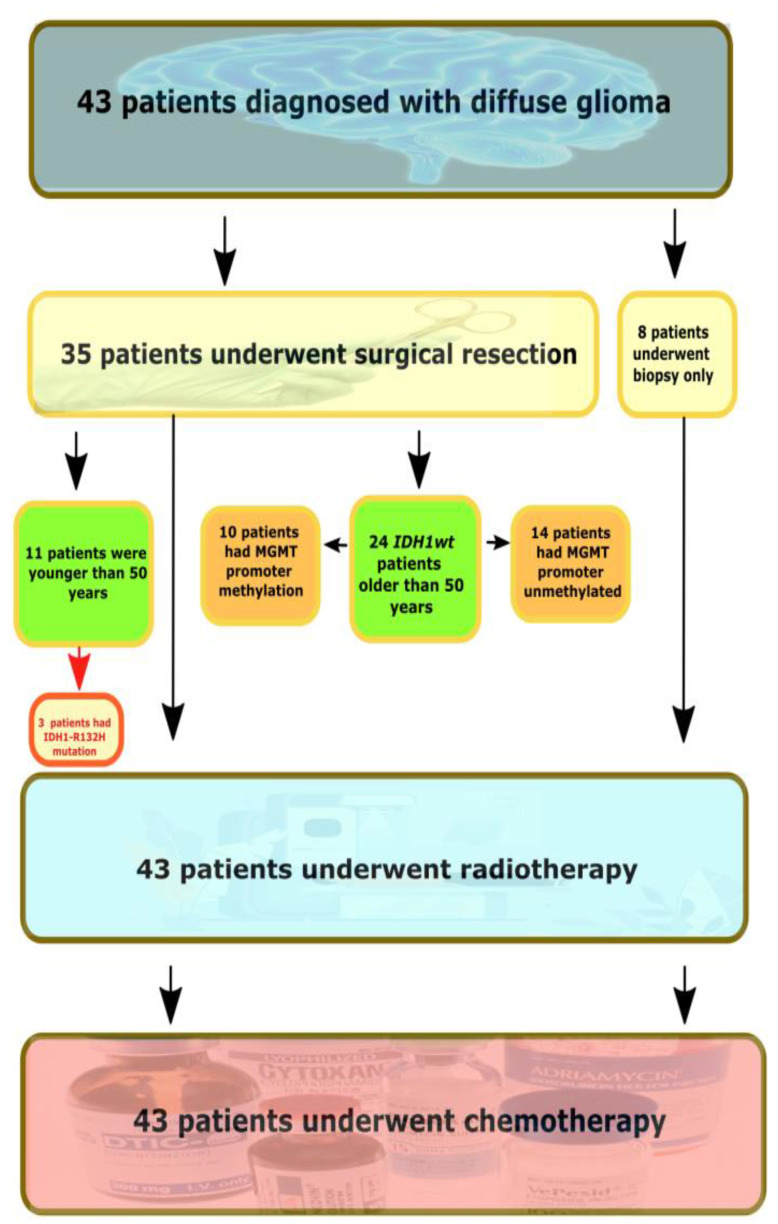
The flow chart of the study. *IDH1wt*—IDH-wildtype tumors.

The majority of tumors were histopathologically diagnosed by WHO instructions as adult-type diffuse gliomas (*n* = 43), while meningioma and hemangiopericytoma samples were included as control samples. The flow chart of the study is presented in Figure 1.

**Figure 2:**
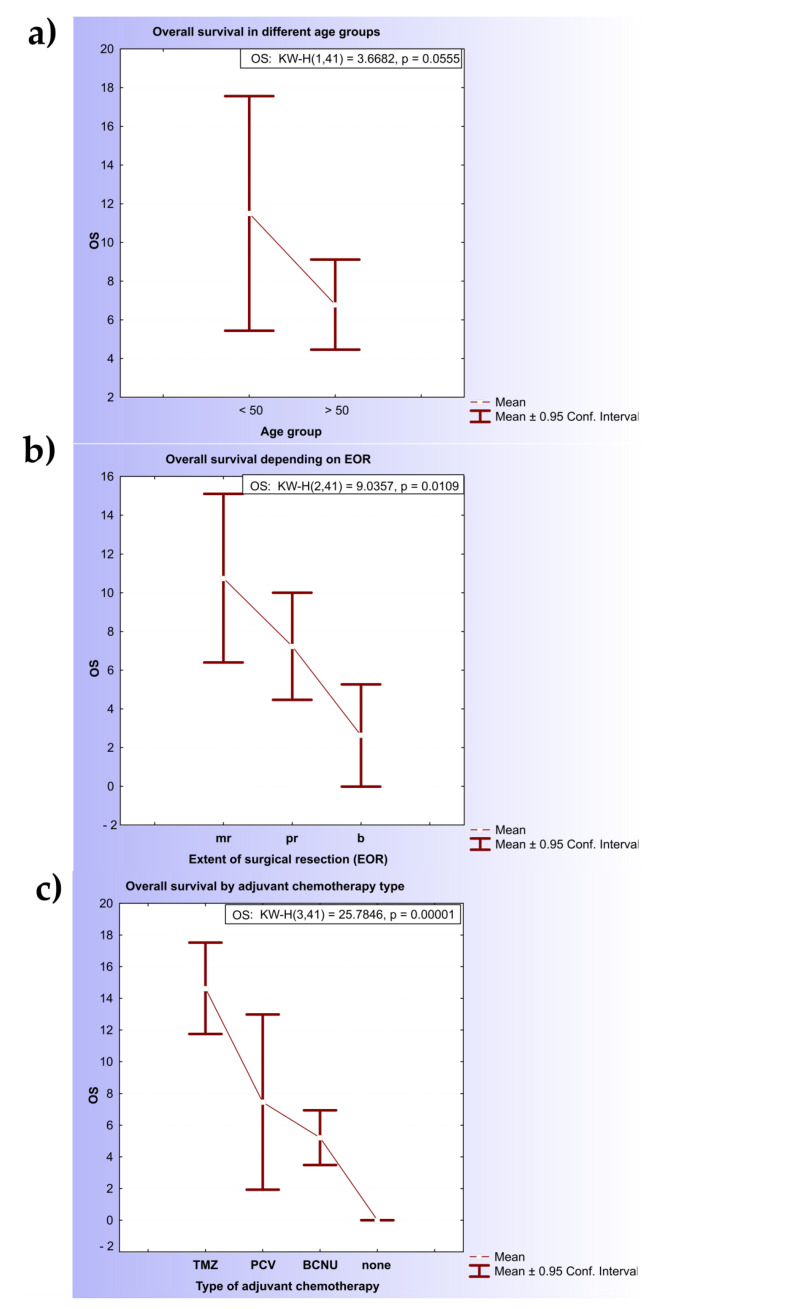
Kaplan–Meier estimates for diffuse glioma patients. (**a**) Kaplan–Meier curves of patients with diffuse glioma: association of age and OS; (**b**) Kaplan–Meier curves of patients with diffuse glioma: association of extension of resection and overall survival; (**c**) Kaplan–Meier curves of patients with diffuse glioma: association of therapy and overall survival. Note: OS—overall survival; TMZ—temozolomide; PCV—procarbazine, lomustine (1-[2-chloroethyl]-3-cyclohexyl-1-chloroethylnitrosourea (CCNU)) and vincristine; BCNU—carmustine.

The clinical characteristics of the patients are shown in Table S1 (Supplementary Materials). The median age was 58.93 years (range: 26–81), 24.4% of the patients were younger than 50 years, and the male-to-female ratio was 1.73. The median overall survival (OS) estimated for patients older than 50 years was significantly shorter (6.78 months; 95% CI, 4.45- 9.11) in comparison with the younger group (11.5 months; 95% CI, 5.44–17.56) (KW-H(1,41) = 3.6682, *p* = 0.055) (Figure 2a). The Kaplan–Meier estimates of overall survival in the two age subgroups were significantly different (*p* = 0.06 by the log-rank test).

Considering the extension of surgical resection (EOR), patients that underwent surgical resection of the primary tumor had significantly longer overall survival (8.94 months; 95% CI, 6.46–11.42) in comparison with patients who had undergone biopsy (only 2.62 months; 95% CI, 0–5.27) (KW-H(2.41) = 9.0357, *p* = 0.0109) (Figure 2b).

Among the different types of adjuvant chemotherapy, TMZ was correlated with the longest OS (14.64 months; 95% CI, 11.75–17.52), followed by PCV (7.45 months; 95% CI, 1.92–12.98) and the BCNU regimen (5.21 months; 95% CI, 3.48–6.94). Statistically significant differences were noted between the TMZ/PCV (KW-H(1.22) = 10.206, *p* = 0.0014) and TMZ/BCNU (KW-H(1.25) = 15.4282, *p* = 0.00009) groups but not PCV and BCNU (Figure 2c).

The clinical characteristics were subjected to a Cox regression analysis (Table 2). The age, extent of surgical resection, and type of adjuvant chemotherapy were significantly associated with overall survival by the univariate Cox regression analysis. An age younger than 50 years and TMZ adjuvant chemotherapy were associated with a better prognosis (hazard ratio below 1), whereas biopsy and the absence of adjuvant chemotherapy were associated with an unfavorable prognosis.

Finally, the correlation coefficients between *MGMT* methylation and diagnosis, chemotherapy type, EOR, and age group indicated the existence of moderate associations (φ_c_ = 0.2649948; φ_c_ = 0.1765330; φ_c_ = 0.2744930; and r_t_ = 0.3750831, respectively).

### 2.2. IDH1/2 Mutation Status

Exon 4 from the *IDH1* gene and *IDH2* gene were successfully amplified by PCR and sequenced by the Sanger method (Section 4 and Supplementary Figures S1–S5).

A Geneious Prime software analysis of the IDH1 exon 4 amplicons detected 71.16 ± 1.88% of untrimmed base sequences that were high-quality and a GC content of ≈40%. A multiple sequence alignment with the reference sequence (NM_005896.4) and an inspection of the Sanger electropherogram at position 395 revealed the presence of the heterozygous G > A missense mutation in three patients (6.97%). This mutation, which causes the substitution from arginine to histidine at codon 132 (IDH1R132H), was found in two tumor samples that were histopathologically classified as oligoastrocytoma and one GBM sample (Figures S1 and S2 in the Supplementary Materials). The mean age of patients with the IDH1R132H mutation (41 ± 6.24 years) was lower than that of the IDH1-wildtype group (60.27 ± 2.05). The overall survival of patients with detected IDH1R132H (17 ± 8.7) was longer in comparison with the IDH1-wildtype subgroup (6.97 ± 0.9) (KW-H(1.41) = 1.4545, *p* = 0.2278).

Similarly, the *IDH2* gene was screened for mutations (Figures S3–S5 in the Supplementary Materials). Sanger sequencing was performed successfully (75.18 ± 12.95% of the high-quality sequences). However, neither the *IDH2R140* nor *IDH2R172* mutations were detected within this study group after multiple sequence alignment with the reference sequence (NM_002168.4).

### 2.3. MSP Evaluation of MGMT Methylation Status

The MSP data were analyzed using the previously reported semi-quantitative approach, which relies on an ImageJ software comparison of the intensities of the methylated (M) and unmethylated (U) MSP bands (Figure S6 in the Supplementary Materials) [23]. In this manner, the *MGMT* methylation status was evaluated in 36 glioma patients and 3 negative control samples (meningioma, hemangiopericytoma, and peripheral blood leukocytes of a healthy individual). There were 17 patients (47.2%) with assigned negative methylation status (unmethylated *MGMT*) and 19 (52.8%) with positive methylation status (methylated *MGMT)*. Among patients with methylated *MGMT*, 7 patients were assigned to the weakly methylated semi-quantitative subgroup (M/U ratio between 0 and 1) and 12 were assigned to the strongly methylated (M/U ratio > 1) subgroup. The significant difference in OS between positively methylated (M/U > 1) and unmethylated (M/U = 0) patients was not detected using either the Kaplan–Meier method (*p* = 0.33 by the log-rank test) or a Cox regression analysis (*p* = 0.33).

The statistical analyses were reduced to a homogenous cohort, considering their clinical characteristics. Given the strong prognostic influence of age, the study group was primarily reduced to the >50 years cohort, as it included a greater number of patients than the <50 years group. Afterward, cases with a performed biopsy as the only type of surgical intervention were excluded from the analyses. This was performed due to a significant difference in the OS of patients with biopsy in comparison with the group of patients with maximal/partial surgical resection of the tumor mass. Finally, the *IDH1R132H-*mutated group of patients was excluded from the analyses, given their longer OS in comparison with the *IDH1*-wildtype group. Following this reduction, clinical data of 17 diffuse glioma patients were left for statistical analyses: 11 with unmethylated (M/U = 0) and 6 with hypermethylated (M/U > 1) semi-quantitative evaluations of *MGMT* methylation status. The mean OS of the hypermethylated group of patients was 8 months, whereas the OS of the unmethylated group was 5 months (KW-H(1.17) = 2.5859, *p* = 0.10). Survival curves were estimated for each group using the Kaplan–Meier method and compared statistically using the log-rank test. The obtained results suggested the improvement of statistical parameters (log-rank = −1.70785, *p* = 0.08766) that were much closer to statistical significance in comparison with the previous analysis of the entire group of patients.

The semi-quantitative *MGMT* methylation status was further subjected to a univariate Cox regression analysis, which suggested the favorable prognosis of the patients with methylated *MGMT*.

### 2.4. qMSP Evaluation of MGMT Methylation Status

**Figure 3:**
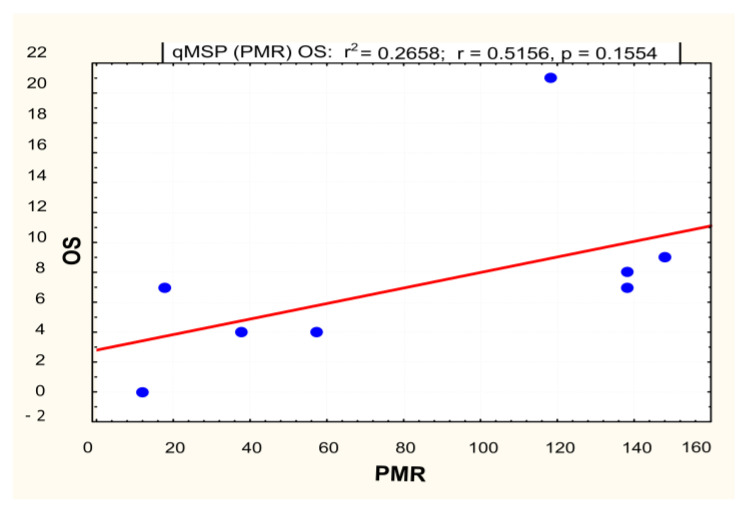
Linear regression model for qMSP for methylated *MGMT* patients (*mMGMT*). Note: OS—overall survival; PMR—percentage of methylated reference.

The level of *MGMT* promoter methylation was quantitatively evaluated by qMSP as the percentage of methylated reference (PMR). According to the previously described method, the PMR value was calculated by dividing the methylated *MGMT/ALUC4* quantity ratio in a sample and the methylated *MGMT/ALUC4* quantity ratio in a fully methylated human genomic DNA control and multiplying by 100. The quantity of 0 (PMR = 0) was assigned to 11 samples with Ct-values above 35. The qMSP result of the control meningioma sample was used to define the initial cut-off value to be used in the discrimination between methylation-positive and methylation-negative samples. A PMR value above zero was detected in 23 tumor samples (54%), which were evaluated as positively methylated. Within the homogenous cohort of 22 patients, 50% (*n* = 11) were evaluated as positively methylated using the PMR = 0 cut-off value. A linear regression analysis that included patients with assigned positive PMR values did not confirm the statistical significance of the relationship between the OS and the quantitative methylation level of the *MGMT* promoter (*p* = 0.15) (Figure 3).

**Figure 4:**
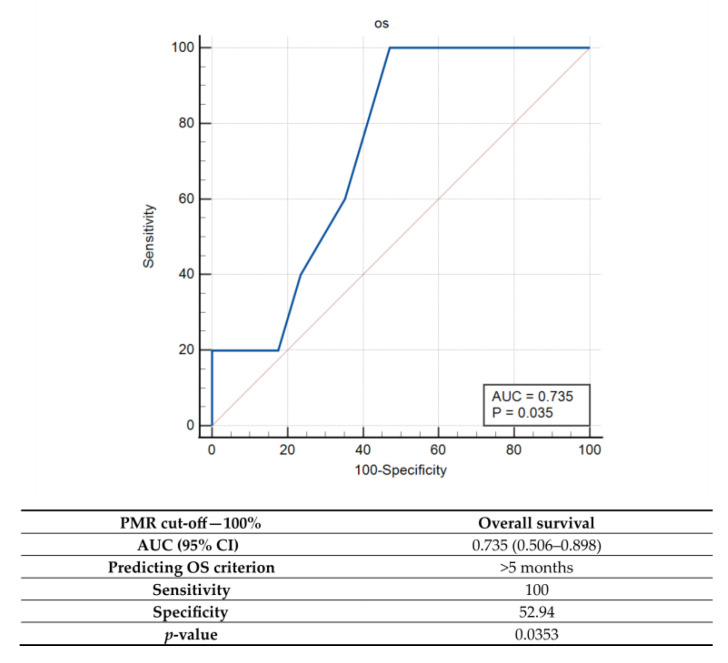
Receiver operator characteristic (ROC) curve analysis for qMSP when cut-off value of PMR = 100%. Note: PMR—percentage of methylated reference, AUC—area under the curve; CI—confidence interval; *p*—probability value.

Since the use of the initial PMR = 0 threshold value did not result in significant differences in OS between *uMGMT* and *mMGMT,* additional cut-off values were explored by the ROC. Among them, the best overall accuracy (AUC) was detected for a PMR = 100% cut-off value, which was characterized by high sensitivity (100%) but low specificity (high rate of false positives) for predicting an OS > 5 months (Figure 4).

The mean overall survival of the *uMGMT* group of patients (*n* = 17) was 5.88 ± 0.88, while the mean overall survival of the *mMGMT* group (*n* = 5) was 10.4 ± 2.68 (KW-H(1.22) = 2.4947, *p* = 0.1142) (Figure S7 in the Supplementary Materials).

However, neither the univariate Cox regression analysis (*p* = 0.23) nor the log-rank comparison of the Kaplan–Meier survival curves (log-rank = 1.52278, *p* = 0.12781) found statistically significant differences in OS between the *uMGMT* and *mMGMT* groups of patients (Figure S8 in the Supplementary Materialsand Table 3).

### 2.5. Combined MSP and qMSP Analyses for Evaluation of MGMT Methylation Status Prognostic Significance

**Figure 5:**
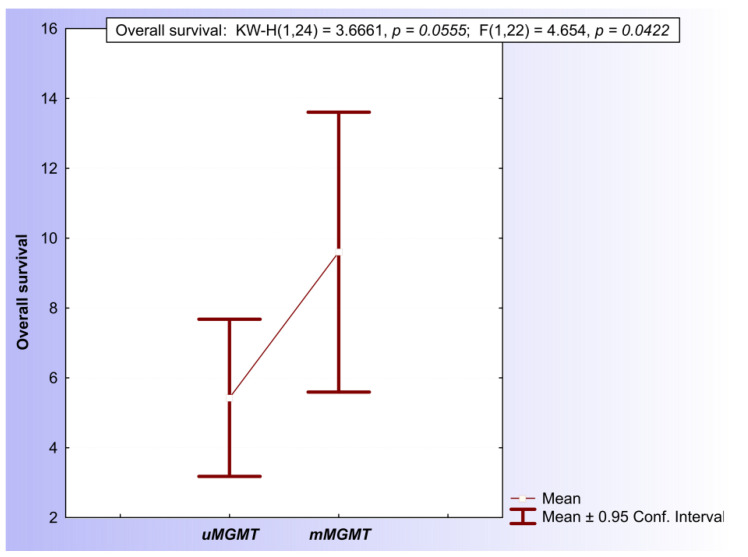
Comparison of mean OS of patients with positive *MGMT* promoter methylation status and negative methylation status.

Following the close inspection of both the MSP and qMSP results, a final conclusion about the *MGMT* promoter methylation status was determined for every patient. A tumor sample was evaluated positively for the methylation of the *MGMT* promoter in the case of either a hypermethylated MSP evaluation (M/U > 1) or a positive qMSP evaluation based on the PMR > 100% cut-off values. Within the GBM subpopulation of patients, there was no difference in OS between the *mMGMT* and *uMGMT* groups (log-rank = 0.597977, *p* = 0.54986) (Figure S9). Within the homogenous cohort of 24 *IDH1/2wt* patients older than 50 years with performed surgical resection of the tumor mass, 10 patients (41,6%) were evaluated positively and 14 were evaluated negatively for the presence of *MGMT* promoter methylation. The mean overall survival of the *mMGMT* group of patients (9.6 ± 1.77 months) was significantly longer in comparison with the *uMGMT* group of patients (5.43 ± 1.04 months) (KW-H(1.24) = 3.6661, *p* ≈ 0.05) (Figure 5).

**Figure 6:**
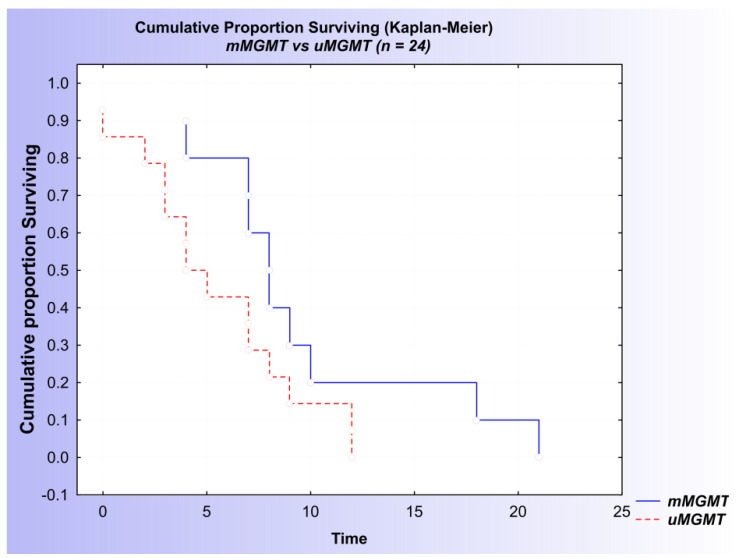
Kaplan–Meier curves of patients with diffuse glioma: association of *MGMT* status and overall survival based on the combined results of the MSP and qMSP analyses.

In addition, both the Kaplan–Meier analysis and the univariate Cox regression analysis suggested a difference in survival between the *mMGMT* and *uMGMT* groups of patients, with a *p*-value proximate to 0.05 (log-rank = −1.76614, *p* = 0.07737) (Figure 6 and Table 4).

**Figure 7:**
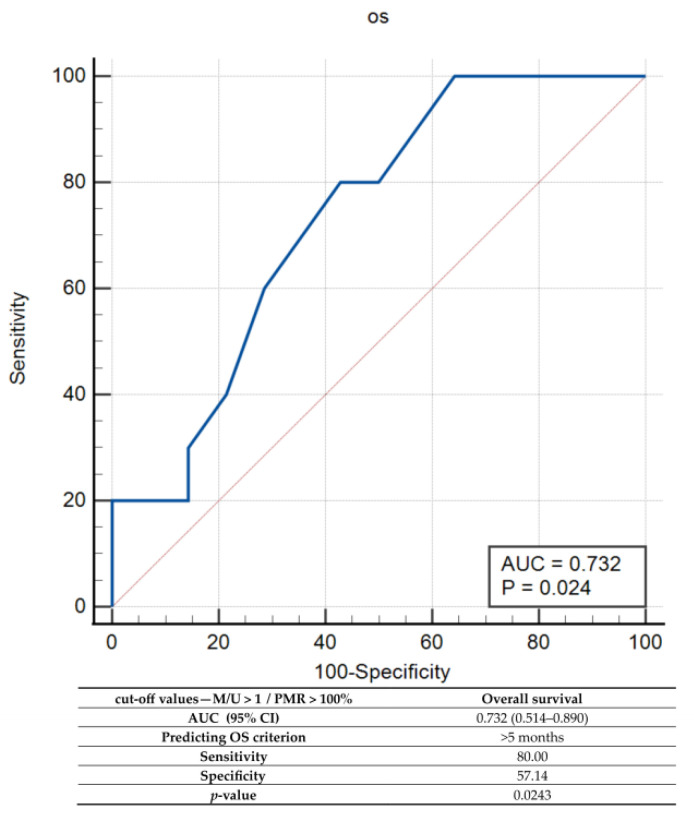
Receiver operator characteristic (ROC) curve analysis for combined MSP and qMSP analyses. Note: PMR—percentage of methylated reference, AUC—area under the curve; CI—confidence interval; *p*—probability value.

Finally, the combined set of MSP/qMSP cut-off values was tested by the ROC and characterized by a sensitivity of 80% and 57.14% specificity for predicting an OS > 5 months (Figure 7).

## 3. Discussion

Despite all the improvements in the standard treatments and all the progress in understanding the molecular pathogenesis of glioma genesis, the median survival of malignant diffuse glioma (and GBM) patients has not substantially improved. This is mainly because these tumors rapidly evolve into radio- and chemoresistant forms that infiltrate the surrounding brain tissue, making complete surgical eradication impossible. Nonetheless, diffuse gliomas are known for extensive intra- and intertumoral and interpatient heterogeneity, which are major obstacles to the improvement of early diagnosis and treatment protocols [24,25]. It is challenging to discriminate genetic alterations that initiate and drive gliomagenesis (the drivers) from those that have been accumulated by chance and are neutral to the process (the passengers) and to interrelate them with the epigenetic alterations that drive changes in the epigenomic environment and modulate gene expression and glioma progression and metastasis [10]. Precision cancer medicine targets distinct genetic and epigenetic features of glioma cells to improve disease management and patient survival. [26,27]. In pursuing such a goal, numerous studies and clinical trials have reported several molecular biomarkers as reliable prognostic and predictive factors of high-grade gliomas and newly diagnosed GBM [28,29].

Among the most important epigenetic biomarkers is the methylation status of the *MGMT* promoter, a widely accepted predictor of prolonged survival upon treatment with alkylating agents such as Temozolomide [30,31,32,33,34]. However, several studies (Jesien-Lewandowicz et al. (2009) on 32 primary GBMs from Polish patients [35]; Kalkan et al. (2015) on 40 primary GBMs from Turkish patients [36]; Ilić et al. (2017) on 62 GBMs from Serbian patients [37]; and Egaña et al. (2020) on 107 high-grade gliomas from Spanish patients) [38], including our previous study (Jovanović et al. (2019) on 25 GBMs from Serbian patients) [22], did not confirm the prognostic and/or predictive value of *MGMT* promoter methylation.

The current study aimed to reinvestigate the prognostic role of the *MGMT* promoter methylation status in 43 samples of diffuse glioma from Serbian patients that were additionally stratified by *IDH1/2* mutation status. The *MGMT* promoter methylation status was investigated in the context of the demographic and clinical data, the choice of method for its assessment, and the IDH1/2 mutation status.

### 3.1. Demographic and Clinical Prognostic Factors

The Southern-Eastern European Registries (SEE); the United States Surveillance, Epidemiology, and Results (SEER); and the Central Brain Tumor Registry of the United States (CBTRUS) define advanced age, male sex, race/ethnicity (with the largest Black–White disparities), and rural residency at diagnosis of brain and other CNS tumors as adversely affecting outcomes [2,3,39]. Despite minor differences in the age limits used to categorize the patients, numerous studies and randomized control trials have found a strong connection between age and the overall survival of high-grade glioma patients [40,41,42]. According to such observations, GBM patients older than 50 years are characterized by significantly shorter overall survival (4.55 months) in comparison with the younger group of patients (*p*= 0.0007, HR= 1.0683, 95% CI–1.0281–1.1101) [43]. The current study further confirms this connection, as the Kruskal–Wallis comparison of the overall survival suggested significant differences in OS between the >50 years (6.78 months) and <50 years (11.5 months) age groups (KW-H(1.41) = 3.6682, *p* = 0.055). Furthermore, the univariate Cox regression analysis confirmed the correlation between the <50 years age group and better prognosis (*p* = 0.03, HR = 0.42). According to the European Association for Neuro-Oncology (EANO) evidence-based guidelines on the diagnosis and treatment of adult-type diffuse gliomas, the evaluation of *MGMT* promoter methylation status is particularly recommended for “elderly or frail patients, to aid in decision-making for the use of temozolomide” [44]. Depending on their *MGMT* methylation status, patients could undergo TMZ adjuvant chemotherapy in the case of positive methylation status or hypofractionated radiotherapy alone in the presence of negative methylation status. Considering that, the present study aimed to explore the prognostic significance of the MGMT promoter methylation status within the >50 years cohort of high-grade glioma patients.

The extent of tumor resection represents another independent prognostic factor that is strongly positively correlated with OS in high-grade glioma [45,46,47,48]. The NOA-04 randomized phase III trial of sequential radiochemotherapy of anaplastic glioma with procarbazine, lomustine, and vincristine or temozolomide recognized the EOR as an important prognosticator [41]. A multivariate Cox regression analysis (*p* = 0.0006) that aimed to depict the prognostic influence of the EOR among GBM patients discovered that the biggest difference was present between biopsy and complete resection (HR = 3.5), followed by biopsy and incomplete (partial) resection (HR = 2.1), and incomplete and complete resection (HR = 1.6). Based on these findings, it would be acceptable to consider patients with performed complete/incomplete resection separately from the patients with performed biopsy alone. The current study confirmed such an assumption by finding significant differences in OS between patients who underwent surgical resection of the primary tumor (8.94 months) and those who had undergone biopsy only (2.62 months) (KW-H(2,41) = 9.0357, *p* = 0.0109). Biopsy was also associated with an unfavorable prognosis by a univariate Cox regression analysis (*p* = 0.02, HR = 2.86). Based on this finding, patients who had undergone biopsy alone were excluded from the statistical analyses considering the evaluation of the prognostic significance of the *MGMT* promoter methylation status.

The adjuvant nitrosourea (BCNU/PCV) and TMZ treatment of high-grade glioma patients represents a well-known positive prognostic factor in comparison with its absence (*p* < 0.001; HR = 1.622) [45]. In addition, the TMZ treatment is associated with significantly longer OS (15.9 months) in comparison with BCNU (11.5 months) [48]. Regarding the prognostic features of adjuvant chemotherapy, our results were in concordance with previous studies. Confirming the superiority of its application in high-grade glioma patients, TMZ was correlated with a significantly longer OS (14.64 months; 95% CI, 11.75–17.52) in comparison with PCV (7.45 months; 95% CI, 1.92–12.98) and the BCNU regimen (5.21 months; 95% CI, 3.48–6.94). This correlation was also detected by a univariate Cox regression analysis in which TMZ adjuvant chemotherapy was associated with a better prognosis (*p* = 0.0018, HR = 0.29), whereas the absence of adjuvant chemotherapy was associated with an unfavorable prognosis (*p* = 0.0023, HR = 28.168).

### 3.2. The MGMT Methylation Status Assessment and the Choice of Method

The most important question that arises in studies, such as this one, with controversial findings, especially in countries with limited resources in health care, such as Serbia, is the choice of the optimal method for routine clinical diagnostics [49]. Methylation-specific PCR (MSP) and real-time MSP (qMSP) methods have been proposed as the most suitable for introduction in clinical practice [50]. In previous studies, we used the least expensive method for *MGMT* methylation status assessment, MSP [22,23,51], whereas in the present study we compared MSP as a semi-quantitative method with real-time MSP (qMSP) as a quantitative method.

MSP represents a widely accepted method for the evaluation of *MGMT* promoter methylation status and is supported by a vast amount of evidence [49]. It relies on the bisulfite conversion of the tumor DNA samples before the PCR amplification of specific CpG dinucleotides within the MGMT promoter region. Using the specifically designed primers, MSP enables the qualitative interpretation of the *MGMT* promoter methylation signal by assessing the amplified sequences via gel electrophoresis. Although MSP provides simple and easy-to-interpret results in the case of strongly methylated and unmethylated signals, earlier studies have emphasized the big drawback regarding the interpretation of faint gel electrophoresis signals, typically referred to as “equivocal”. Such signals are often left for the subjective interpretation of researchers. Furthermore, it was suggested that the plain dichotomization of methylation signals could be misleading and inadequate for prognostication. For example, a “dose-response” trend was noted, wherein the level of MGMT methylation correlated with the survival of patients [49]. Semi-quantitative approaches could increase the sensitivity of the MSP method by classifying the patients into three semi-quantitative groups (unmethylated, weakly methylated, and strongly methylated), depending on the ratios of the fluorescence signal intensities of the methylated and unmethylated *MGMT* MSP products on a gel (M/U ratio). In previous studies, our research group recognized the potential of the semi-quantitative MSP method for introduction into the clinical routine and explored several semi-quantitative MSP method approaches [22,23].

Quantitative MSP (qMSP) shares the basic principle with the conventional MSP assay, with the addition of the use of Real-Time PCR technology for the relative quantification of the *MGMT* promoter methylation. As in conventional MSP, most qMSP assays usually cover the same 11 CpG sites that were shown to be relevant. The amplification of a part of the ALU element (ALU C4) is used for the normalization, and commercial unmethylated (bisulfite-treated normal leukocyte DNA) and methylated (bisulfite-converted in vitro methylated human DNA) DNA is used for control reactions. Moreover, the use of DNA isolates from meningioma samples is recommended for the cut-off/methylation threshold signal definition [52]. Despite the improvement that came with the quantitative MSP assessment of the *MGMT* methylation status, the term “equivocal” or “gray zone” has also been applied to the qMSP method [49]. It refers to the range of values in the vicinity of the cut-off value, where there is high uncertainty in defining the samples as “methylated” [49].

The problem in defining the optimal cut-off values remains regardless of the type of MSP approach. This task is particularly demanding due to the existence of the “partially methylated” samples, wherein not all CpG sites are methylated. As “methylated *MGMT*” PCR primer pairs were designed for the detection of a fully methylated (all 11 CpG sites) promoter region, both MSP and qMSP classify such samples mostly to the “unmethylated” group, thus lowering their sensitivity and prognostic values. Despite the limitations, the majority of available data give priority to the qMSP assay, emphasizing its clinical utility and good balance between reliability, availability, and cost. Following the above, in the present study, the evaluation of *MGMT* promoter methylation status was primarily assessed by the qMSP method, whereas semi-quantitative conventional MSP served as an auxiliary approach to confirm and explore the obtained results. Thus, a tumor sample was evaluated positively for the methylation of the *MGMT* promoter in the case of either a hypermethylated MSP evaluation (M/U > 1) or a positive qMSP evaluation based on the PMR > 100% cut-off values. In concordance with the mentioned randomized control trials, the statistical analyses regarding the combined MSP/qMSP evaluation of the *MGMT* promoter methylation status have indicated its prognostic importance within the Serbian population of diffuse glioma patients (KW-H(1.24) = 3.6661, *p* ≈ 0.05) [19,20]. However, our results suggest its significance only within the uniform cohort of *IDHwt* elderly patients (>50 years) with performed surgical resection of the tumor mass. Such findings support previous reports of the importance of MGMT promoter methylation status evaluation as an aid in decision making related to the use of chemotherapy in elderly patients [44].

### 3.3. IDH1/2 Mutations

*IDH 1* and *2* missense mutations represent initiating genetic events and are one of the key molecular features of low-grade gliomas (70–80%) and secondary GBM [53,54,55]. The most important *IDH* mutation in GBM is *IDH1-R132H,* which was introduced in the 2016 WHO Classification for Tumors of the Central Nervous System as the molecular marker of low-grade glioma (LGG) and secondary GBM [56]. Mutant *IDH1* was shown to promote the glioma cytosine–phosphate–guanine (CpG) island methylator phenotype (G-CIMP) in gliomas, which are often characterized by the epigenetic silencing of *MGMT* [57,58]. As a consequence, the response to alkylating agent treatment (TMZ or nitrosoureas) is more favorable among secondary GBM patients, which results in a significant difference in OS between these GBM subtypes (secondary GBM—31 months, primary GBM—15 months) [6]. Due to the described interconnection, the combined detection of *IDH1* mutation and *MGMT* promoter methylation surpass their individual significance in the prediction of survival in GBM patients [59,60,61]. The longest OS was observed among patients with a present *IDH1-R132H* mutation and a positive *MGMT* promoter methylation status [60]. Thus, the initial step for exploring the prognostic role of the *MGMT* promoter methylation status in the present study was the detection of *IDH1/2* mutations in tumor samples by Sanger sequencing. This was performed to discriminate *IDH1wt* from *IDHmut* samples and identify the *IDHwt* homogenous cohort of high-grade glioma patients. For the detection of *IDH1* and *IDH2* mutations, two different sets of primers were designed to amplify exon 4 of *IDH1* and exon 4 of *IDH2.* A software analysis of the Sanger electropherogram data showed a satisfactory percentage of untrimmed bases in sequences that were high-quality (71.16 ± 1.88% for *IDH1* amplification and 75.18 ± 12.95% for *IDH2* amplification). After the alignment of the obtained sequences with the reference sequence, three patients were evaluated to be positive for the *IDH1-R132H* mutation, and none of the samples were positive for *IDH2 (R140* and *R172)* mutations. Considering the sample size within this study group and the previous reports of the very low frequency of *IDH2mut* in GBM samples, such a result was expected [62]. The histopathological diagnoses of *IDH1mut* patients were oligoastrocytoma (two patients) and glioblastoma (one patient). This result is in concordance with the observation that 98% of oligoastrocytoma samples carry the *IDH1-R132H* mutation, thus confirming their previous histopathological classification [63]. The observed low frequency of *IDHmut* (secondary GBM) is in agreement with the previously reported low frequency of secondary GBM cases (5%) [53]. Besides the appearance of significant differences in OS, it was previously documented that the majority (90%) of *IDH1-R132H* mutations occur within GBM patients younger than 55 years [64]. With longer observed OS within *IDHmut* patients (17 months to 7.7 months) as well as younger patients (41 ± 10.8 years), this study further confirmed such observations, despite its small sample size. As mentioned before, the *IDH1-R132H* mutation is strongly correlated with positive *MGMT* promoter methylation status (57% cases) [65]. All of the *IDH1-R132H*-positive patients were also positively evaluated for *MGMT* promoter methylation by both MSP and qMSP methods. Furthermore, the present study confirms the correlation of the IDH1mut/MGMTmet genotype and longer OS in comparison with other combinations in the IDH1/MGMT two-gene predictor model [60].

## 4. Materials and Methods

### 4.1. Tumor Specimens

A total of 45 patients pathologically diagnosed with diffuse glioma at the Neurosurgery Clinic at the Clinical Centre of Niš were involved in this study. Glioma specimens were obtained from patients who underwent surgery from June 2013 to December 2019 at the Neurosurgery Clinic, University of Niš, Serbia, with written inform consent and approval from the Ethics Committee from the Clinical Centre and Faculty of Medicine (permission No. 01-2113-10, 1/4/2013). Glioma samples were snap-frozen/collected in RNAlater^®^ (Qiagen, Hilden, Germany) and stored at −80 °C. The clinical characteristics of patients are shown in Table S1 (Supplementary Materials).

### 4.2. DNA Isolation and Bisulfite Conversion

The extraction of genomic DNA was performed using a QIAamp^®^ DNA Mini Kit (Qiagen, Hilden, Germany) and 25 mg of an FF (fresh-frozen) sample. A total of 2 µg of genomic DNA was modified by sodium bisulfite using an EpiTect^®^ Bisulfite Kit (Qiagen, Hilden, Germany) for the FF DNA sample. A BioSpec–nano UV–VIS Spectrophotometer (Shimadzu, Japan) was utilized for the determination of the quantity and quality of the isolated DNA and bisulfite-converted samples. Isolated DNA samples were inspected for degradation by electrophoresis on 2% agarose gel.

### 4.3. Determination of IDH1 and IDH2 Mutation Status

The *IDH1* and *IDH2* mutation status in glioma samples was detected by polymerase chain reaction (PCR) followed by DNA sequencing. DNA fragments spanning exon 4 of the *IDH1* and *IDH2* genes were amplified by PCR with following primers: *IDH1*, forward 5′-CCATCACTGCAGTTGTAGGTT-3′, reverse 5′-CATACAAGTTGGAAATTTCTGG-3′; IDHR132H, forward 5′-AATGAGCTCTATATGCCATCACTG-3′, reverse 5′-TTCATACCTTGCTTAATGGGTGT-3′; *IDH2*, forward 5′-CAAGCTGAAGAAGATGTGGAA-3′, reverse 5′-CAGAGACAAGAGGATGGCTA-3′.

The PCR products of the *IDH1* gene were generated in a 25 µL reaction mixture including 1 × reaction buffer B1 with 2 mM MgCl_2_ (Solis BioDyne OU, Tartu, Estonia), the appropriate forward and reverse primers (10 pM), 0.2 µM dNTP mix, 1U of FIREPol^®^ polymerase (Solis BioDyne OÜ, Tartu, Estonia), and 100 ng of template DNA. The amplification reaction was carried out in a Mastercycler^®^ Gradient (Eppendorf, Wien, Austria) with an initial denaturation step at 94 °C for 5 min, followed by 35 cycles consisting of 94 °C for 30 s, 54 °C (57 °C for IDHR132H) for 30 s, and 72 °C for 30 s.

The *IDH2* gene was amplified in different conditions. A 25 µL reaction mixture contained 1 × PCR buffer with 1.5 mM MgCl_2_ (Qiagen, Hilden, Germany), the appropriate forward and reverse primers (10 pM), 0.2 µM dNTP mix, 1U of HotStarTaq^®^ polymerase (Qiagen, Hilden, Germany), and 100 ng of template DNA. The amplification reaction was performed in a Mastercycler^®^ Gradient (Eppendorf, Wien, Austria) with an initial denaturation step at 95 °C for 15 min, followed by 35 cycles consisting of 94 °C for 30 s, 61 °C for 1 min, and 72 °C for 1 min and final cycle of extension at 72 °C for 1 min

All PCR reactions were performed in duplicate. The PCR products were checked by agarose gel electrophoresis and sequenced.

### 4.4. Sanger Sequencing

For the detection of *IDH1* and *IDH2* mutations, the samples were directly sequenced with a Big Dye Terminator v3.1 Cycle Sequencing Kit (Applied Biosystems, Waltham, MA, USA) on a 3130 Genetic Analyzer (Applied Biosystems, Waltham, MA, USA). The primers used for the sequencing were the same as those used for PCR. The PCR products were purified to eliminate unincorporated primers and dNTPs using the Big-Dye XTerminator Purification Kit and then subjected to separation by the 3130xl GeneGenetic Analyzer (Applied Biosystems, Waltham, MA, USA). The results were analyzed and compared to reference sequences collected from the GenBank *IDH1* and *IDH2* sequences (IDH1: NM_005896.2 and IDH2: NM_002168.2, respectively).

### 4.5. Determination of MGMT Promoter Methylation Status by MSP

The levels of methylation of the MGMT promoter in the glioma samples were determined by MSP using 100 ng of bisulfite-treated DNA and specific primers: methylated *MGMT* promoter *(M)*, forward 5′-TTTCGACGTTCGTAGGTTTTCGC-3′, reverse 5′-GCACTCTTCCGAAAACGAAACG-3′, unmethylated *MGMT* promoter *(U)*, forward 5′-TTTGTGTTTTGATGTTTGTAGGTTTTTGT-3′, reverse 5′-AACTCCACACTCTTCCAAA AACAAAACA-3′ [15,52]. PCR products were generated in a 20 µL reaction mixture including 1 × PCR buffer with 1.5 mM MgCl_2_ (Qiagen, Hilden, Germany), the appropriate forward and reverse primers (10 pM), 0.2 µM dNTP mix, 1U of HotStarTaq^®^ polymerase (Qiagen, Hilden, Germany), and 100 ng of bisulfite-converted template DNA. The MSP was conducted in a total volume of 20 µL containing 1 × PCR buffer with 1.5 mM MgCl_2_ (Qiagen, Hilden, Germany), the appropriate forward and reverse primers (10 pM), 0.2 µM dNTP mix, 1U of HotStarTaq^®^ polymerase (Qiagen, Hilden, Germany), and 100 ng of bisulfite-converted template DNA. The amplification reaction was carried out in a Mastercycler^®^ Gradient (Eppendorf, Wien, Austria) using the following program: 95 °C for 15 min; then 35 cycles of 95 °C for 50 s, 59 °C for 50 s, and 72 °C for 50 s; and a final extension at 72 °C for 10 min. Control PCR reactions were performed using methylated bisulfite-converted and unmethylated bisulfite-converted human DNA (EpiTect^®^ PCR Control DNA set (Qiagen, Hilden, Germany)). DNA extracted from peripheral blood leukocytes and meningiomas was used as negative controls. All PCR reactions were performed in duplicate. The evaluation of methylation status was conducted as previously described by Jovanović et al. and Christians et al. [21,66]. The level of methylated DNA was expressed as the ratio of fluorescence intensities of the corresponding methylated (M) and unmethylated (U) signals (M/U ratio). In concordance with the mentioned studies, ImageJ software was used for the processing of the 2% agarose gel images and the calculation of the fluorescence intensities of the amplified PCR products [22,66].

### 4.6. Determination of MGMT Promoter Methylation Status by qMSP

The qPCR was performed in an AriaMx qPCR machine (Agilent Technologies, Santa Clara, CA, USA) using a QuantiNova SYBR^®^ Green PCR kit (Qiagen, Hilden, Germany) according to the modified amplification protocol presented by Håvik et al. [52]. A 20 µL reaction mixture included: 1 × QuantiNova Sybr^®^ Green master mix, 1 × QN ROX^TM^ reference dye, the appropriate forward and reverse primers (10 pM), and 100 ng of bisulfite-converted template DNA. The program for qPCR was the following: 95 °C for 2 min, then 35 cycles of 95 °C for 5 s and 60 °C for 11 s. All PCR reactions were performed in duplicate. The methylation levels of the samples were estimated relative to the methylated and bisulfite-converted control DNA (Qiagen, Hilden, Germany) using the 2^−∆∆Ct^ quantification approach. In addition to the methylated *MGMT* promoter sets of the primers, control ALU–C4 primers were used for the purpose of normalization: forward 5′-GGTTAGGTATAGTGGTTTATATTTGTAATTTTAGTA-3′ and reverse 5′-ATTAACTAAACTAATCTTAAACTCCTAACCTCA-3′.

The PMR value was calculated by dividing the methylated MGMT/ALUC4 relative quantity ratio in a sample and the methylated MGMT/ALUC4 relative quantity ratio in the fully methylated human genomic DNA control and multiplying by 100 [52]. A threshold value for scoring methylation-positive samples was defined based on the qMSP result in meningiomas, which had PMR values of zero [52].

### 4.7. Statistical Analysis

Statistical analyses were performed using the SPSS 16.0 software package (IBM Corp., Armonk, NY, USA). The patient analysis included gender, age, Karnofsky performance status, type of resection, therapy, and the genetic or expression status of the marker gene. Overall survival (OS) was measured from the date of surgery to the date of death or last follow-up. OS curves were estimated using the Kaplan–Meier method and compared with a univariate log-rank test. A receiver operator characteristic (ROC) curve analysis was used to evaluate the performance of the diagnostic tests. Cramer’s V and tetrachoric correlation were used to analyze the correlations between the *MGMT* promoter methylation status and the clinical characteristics. The tetrachoric correlation was calculated for binary categorical variables (gender and age group), and Cramer’s V correlation was calculated for nominal categorical variables (the extent of resection, chemotherapy type, and diagnosis).

## 5. Conclusions

Genuine efforts to understand the molecular pathogenesis of malignant diffuse gliomas with the application of novel sequencing and in-depth analysis technologies, unfortunately, have brought small improvements in the diagnosis, treatment, and survival of diffuse glioma patients, which is expressed in terms of months. The epigenetic profiling of diffuse gliomas might be a path towards novel insights into gliomagenesis and metastasis, more precise glioma stratification, and advanced epigenetic treatments. As the most prominent epigenetic alteration in diffuse glioma and a commonly accepted predictive marker, *MGMT* promoter methylation sometimes puzzles researchers and neuro-oncologists. The diversity of methods, cut-off values “in the eyes” of researchers, patient- and tumor- heterogeneity, and sizes of cohorts creates discrepancies in MGMT methylation status studies. The combination of two methods, MSP and qMSP, within the homogenous cohort (*IDH1/2wt* patients older than 50 years and with surgically resected glioma) gave significant relevance to the prognostic value of the *MGMT* promoter methylation status in Serbian diffuse glioma patients. However, these findings should be verified on a larger collection of samples. Obviously, neuro-oncologists should be cautious when deciding on a therapeutic strategy based on *MGMT* methylation status alone. The simultaneous assessment of two or more markers is highly recommended before making a final decision on treatment and patient prognosis.

## Figures and Tables

**Table 1:** List of brain tumor samples included in the study.

Diagnosis	ICD-O	Number of Patients (*n* = 45)
Glioblastoma	9440/3	37
Astrocytoma anaplasticum	9401/3	1
Oligodedroglioma anaplasticum	9451/3	1
Oligoastrocytoma anaplasticum	9382/3	1
Oligoastrocytoma	9382/3	3
Hemangiopericytoma	9150/1	1
Meningioma	9530/0	1

Note: ICD-O—International Classification of Diseases for Oncology code.

**Table 2:** Univariate Cox regression analysis for different clinical prognostic factors in diffuse gliomas.

Prognostic Factors	*p*-Value	Hazard Ratio (HR)	95% CI (HR)
Age (continuous variable)	0.0084	1.0427	1.0108 to 1.0757
<50 years	0.0314	0.4155	0.1866 to 0.9248
The extent of surgical resection			
Biopsy	0.0211	2.8585	1.1710 to 6.9778
Maximal + partial resection	0.1622	0.6005	0.2937 to 1.2277
Type of adjuvant chemotherapy			
TMZ	0.0018	0.2880	0. 1316 to 0.6303
None	0.0023	28.1680	3.2848 to 241.5466

Note: *p*–Value—probability value; HR—hazard ratio; CI—confidence interval.

**Table 3:** Univariate Cox regression analysis for *MGMT* methylation status determined by qMSP.

*MGMT* Promoter Methylation Status	*p*-Value	Hazard Ratio (HR)	95% CI (HR)
Methylated	0.23	0.5160	0.1728 to 1.5409

Note: *p*—probability value; HR—hazard ratio; CI—confidence interval.

**Table 4:** Univariant Cox regression analysis of *MGMT* methylation status determined by combined MSP and qMSP analyses.

*MGMT* Methylation Status	*p*-Value	Hazard Ratio (HR)	95% CI (HR)
Methylated	0.11	0.4982	0.2080 to 1.1933

Note: *p*—probability value; HR—hazard ratio; CI—confidence interval.

## Supplementary Materials

The following supporting information can be downloaded at https://www.mdpi.com/article/10.3390/ijms232113034/s1.
