# Supplementary material for: The Significance of MGMT Promoter Methylation Status in Diffuse Glioma
Source: Int J Mol Sci. 2022 Oct 27;23(21):13034. doi: 10.3390/ijms232113034 (PMC9654114; doi:10.3390/ijms232113034)
Supplement: Supplementary file 1 [file ijms-23-13034-s001.zip › ijms-1969427-supplementary.pdf]

For Supplementary files:

Table S1. The clinicopathological characteristics of patients.

Patient Number	Age	Gender	Diagnosis	Grade (WHO)	Chemotherapy	Extent of surgical resection	Overall survival (OS) (months)	MGMT promoter methylation status (MSP/qMSP)	IDH1/2 mutation
**1**	35	M	GBM	4	TMZ	mr	80+	Negative	Negative
**2**	66	M	GBM	4	PCV	mr	8	Positive	Negative
**3**	57	M	GBM	4	PCV	mr	5	Negative	Negative
**4**	60	M	GBM	4	BCNU	mr	4	Negative	Negative
**5**	36	M	GBM	4	PCV	mr	1	Negative	Negative
**6**	54	F	GBM	4	BCNU	mr	7	Positive	Negative
**7**	68	M	GBM	4	BCNU	mr	10	Positive	Negative
**8**	74	M	GBM	4	PCV	pr	7	Negative	Negative
**9**	40	M	GBM	4	TMZ	mr	13	Positive	Negative
**10**	66	M	GBM	4	TMZ	mr	12	Negative	Negative
**11**	47	M	GBM	4	TMZ	pr	9	Positive	Negative
**12**	62	F	GBM	4	TMZ	pr	18	Positive	Negative
**13**	65	M	GBM	4	PCV	pr	7	Positive	Negative
**14**	63	F	GBM	4	none	b	0	Negative	Negative
**15**	49	M	GBM	4	TMZ	mr	19	Negative	Negative
**16**	52	F	Oligodedroglioma anaplasticum	3	BCNU	mr	0	Negative	Negative
**17**	75	M	GBM	4	none	b	0	Positive	Negative
**18**	77	M	GBM	4	none	b	0	Positive	Negative
**19**	72	F	Oligoastrocytoma	2	none	pr	0	Negative	Negative
**20**	58	M	GBM	4	BCNU	pr	4	Negative	Negative
**21**	73	F	Astrocytoma anaplasticum	3	none	b	0	Positive	Negative
**22**	63	F	GBM	4	PCV	pr	7	Positive	Negative
**23**	50	F	GBM	4	TMZ	mr	12	Negative	Negative
**24**	63	M	GBM	4	BCNU	b	6	Positive	Negative
**25**	69	F	GBM	4	BCNU	pr	4	Positive	Negative
**26**	57	F	GBM	4	PCV	b	3	Positive	Negative
**27**	53	F	GBM	4	BCNU	pr	2	Positive	Negative
**28**	44	M	Oligoastrocytoma anaplasticum	3	PCV	mr	1	Positive	Positive (IDH1-R132)
**29**	46	F	GBM	4	TMZ	mr	14	Negative	Negative
**30**	80	F	GBM	4	BCNU	pr	3	Negative	Negative
**31**	50	F	Oligoastrocytoma	2	PCV	mr	31	Positive	Positive (IDH1-R132)
**32**	65	M	GBM	4	BCNU	pr	8	Positive	Negative
**33**	58	M	GBM	4	TMZ	b	8	Negative	Negative
**34**	29	M	GBM	4	TMZ	mr	19	Positive	Positive (IDH1-R132)
**35**	55	M	GBM	4	TMZ	pr	21	Positive	Negative
**36**	67	M	GBM	4	PCV	pr	8	Negative	Negative
**37**	69	M	GBM	4	PCV	b	4	Positive	Negative
**38**	81	M	GBM	4	BCNU	pr	4	Positive	Negative
**39**	71	F	GBM	4	BCNU	pr	3	Negative	Negative
**40**	73	M	GBM	4	BCNU	pr	9	Negative	Negative
**41**	43	M	GBM	4	TMZ	mr	16	Positive	Negative
**42**	26	M	Oligoastrocytoma	2	none	mr		Negative	Negative
**43**	73	M	GBM	4	BCNU	pr	9	Positive	Negative
**44**	61	F	Hemangiopericytoma	1-2	none	mr		Negative	Negative
**45**	67	F	Meningioma	1-2	none	mr		Negative	Negative

Note: **F**- female; **M**- male; **GBM** – glioblastoma; **1-4** – glioma grade; **TMZ** – temozolomide; **PCV** - procarbazine, lomustine (1-[2-chloroethyl]-3-cyclohexyl-1-chloroethylnitrosourea (CCNU)) and vincristine; **BCNU** – carmustine; **mr** – maximal resection; **pr**- partial resection; **b**-biopsy.

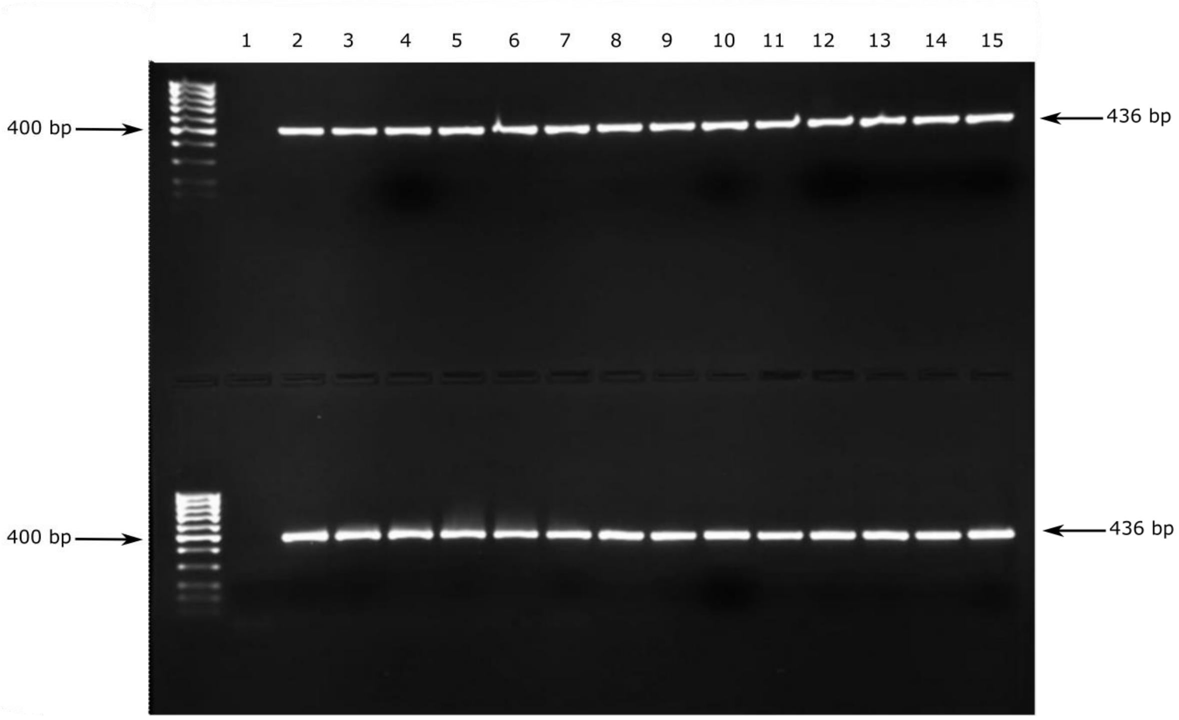

Figure S1. PCR amplification of exon 4 of *IDH1* gene.

1-DNA standard 100 bp ladder; 2-16 -diffuse glioma samples.

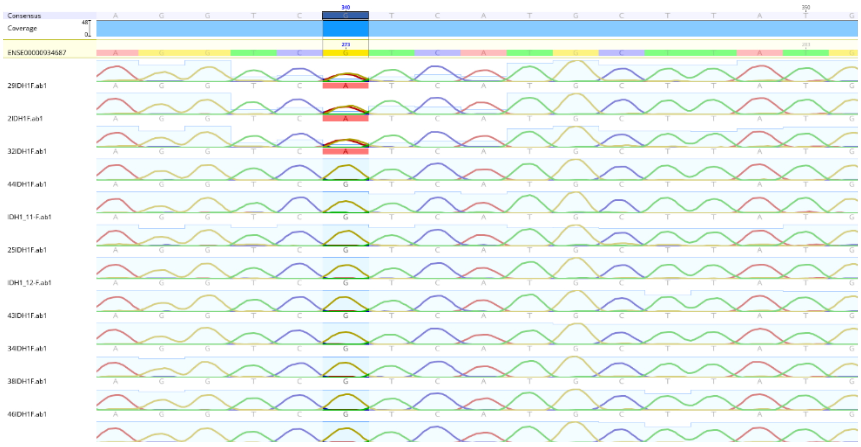

Figure S2. Determination of *IDH1R132*H mutation in diffuse glioma samples.

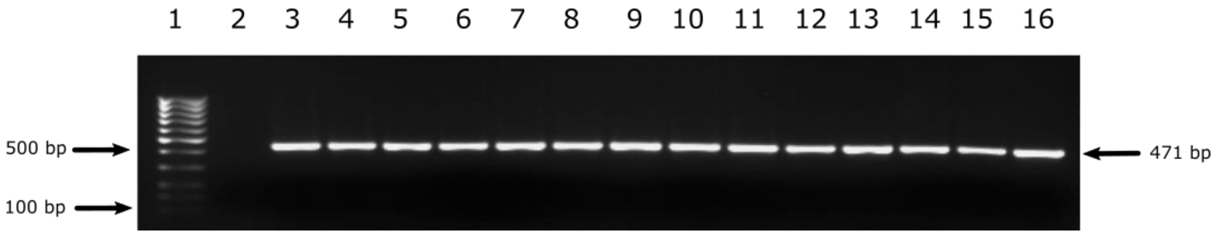

Figure S3. PCR amplification of exon 4 of *IDH2* gene.

1-DNA standard 100 bp ladder; 2-16 -diffuse glioma samples.

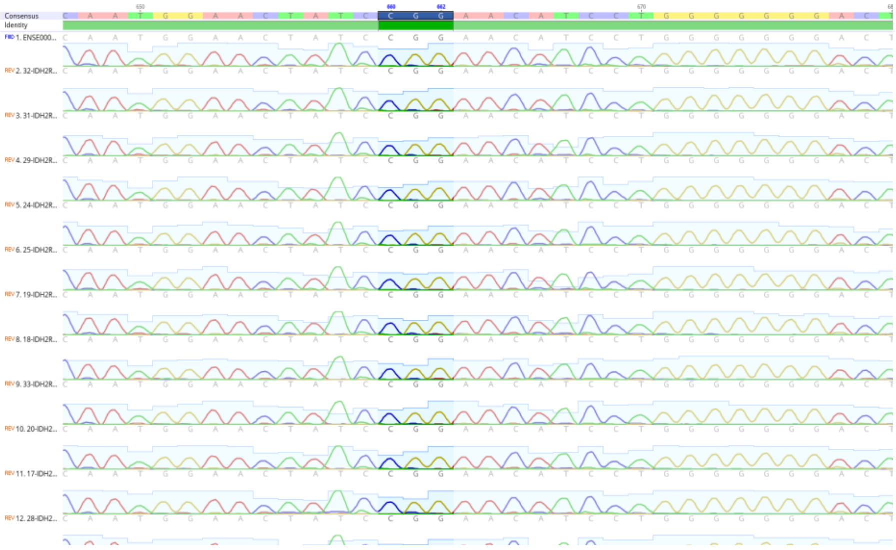

Figure S4. Screening of IDH2 sequence for IDH2R140H mutation in diffuse glioma samples.

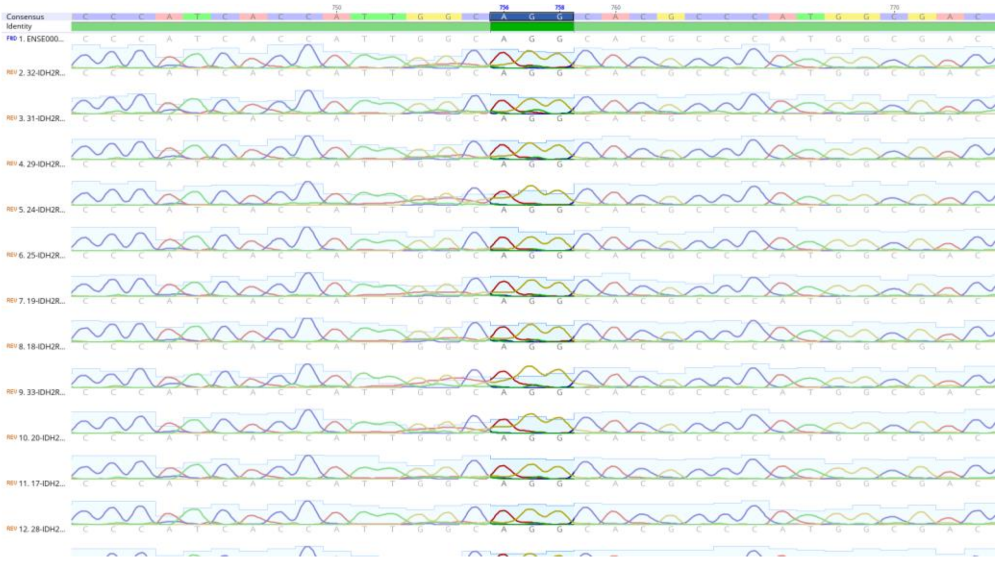

Figure S5. Screening of *IDH2* sequence for *IDH2*R172H mutation in diffuse glioma samples.

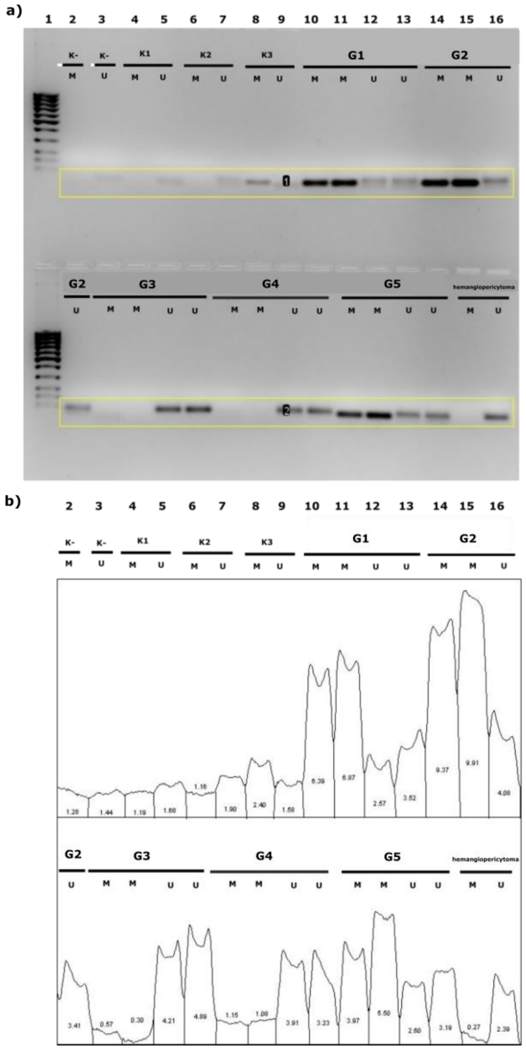

Figure S6. Determination of the methylation status of MGMT promoter in diffuse glioma by methylation-specific polymerase chain reaction (MSP).

(a) Electroforesis on agarose gel of MSP products; (b) ImageJ sotware analysis of intensi-ties of MSP bands.

1-DNA standard 100 bp ladder; 2-16 -diffuse glioma samples in upper and lower gel; K-negative control; K1 - unmethylated human control DNA; K2 -unmethylated and bisulfite-converted human control DNA; K3 -methylated and bisulfite-converted human control DNA; M - polymerase chain reaction (PCR) reaction with primers specific for methylated MGMT promoter; U -PCR reaction with primers specific for unmethylated MGMT promoter; GBM 1-5 - bisulfite-converted DNA from glioma samples.

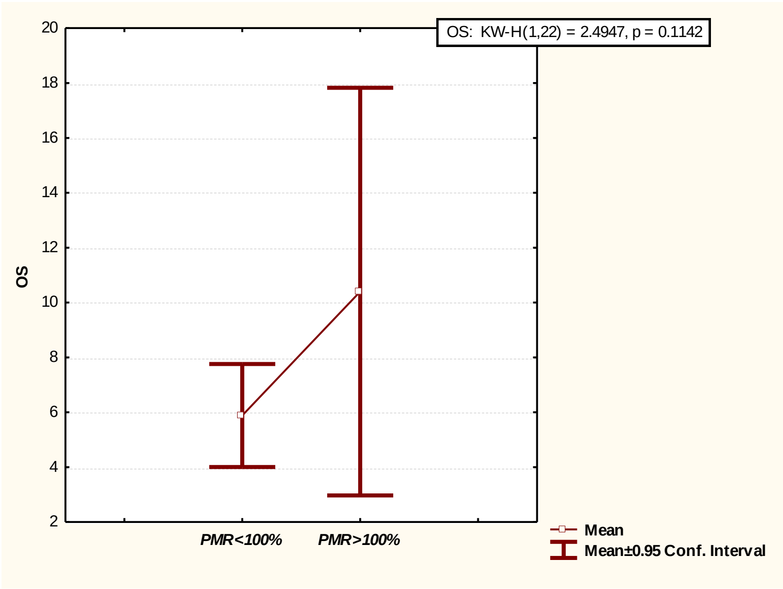

Figure S7. Comparison of mean OS of patients with positive MGMT promoter methylation status (PMR>100%) and negative methylation status (PMR<100%)

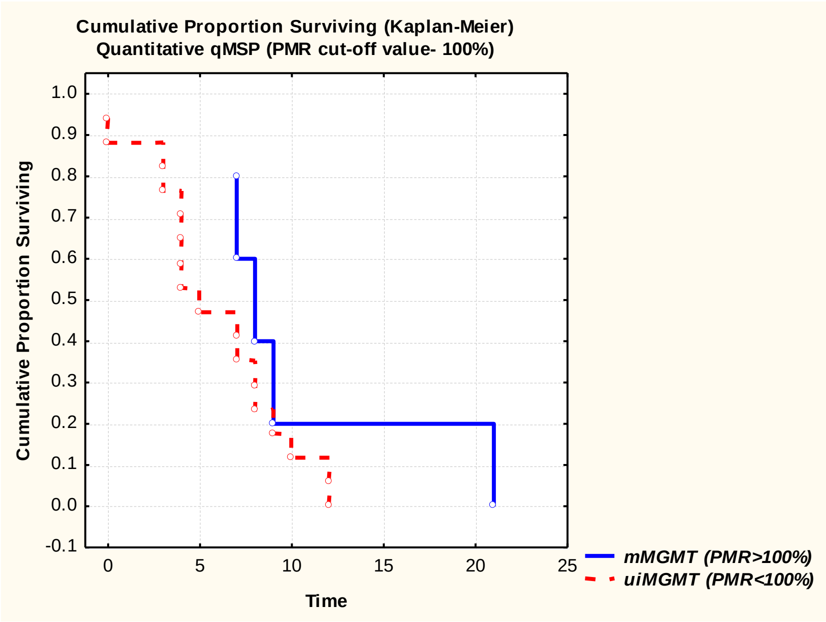

Figure S8. Kaplan-Meier curves of patients with diffuse glioma – association of MGMT status estimated by qMSP and overall survival, considering the PMR=100% as cut-of value

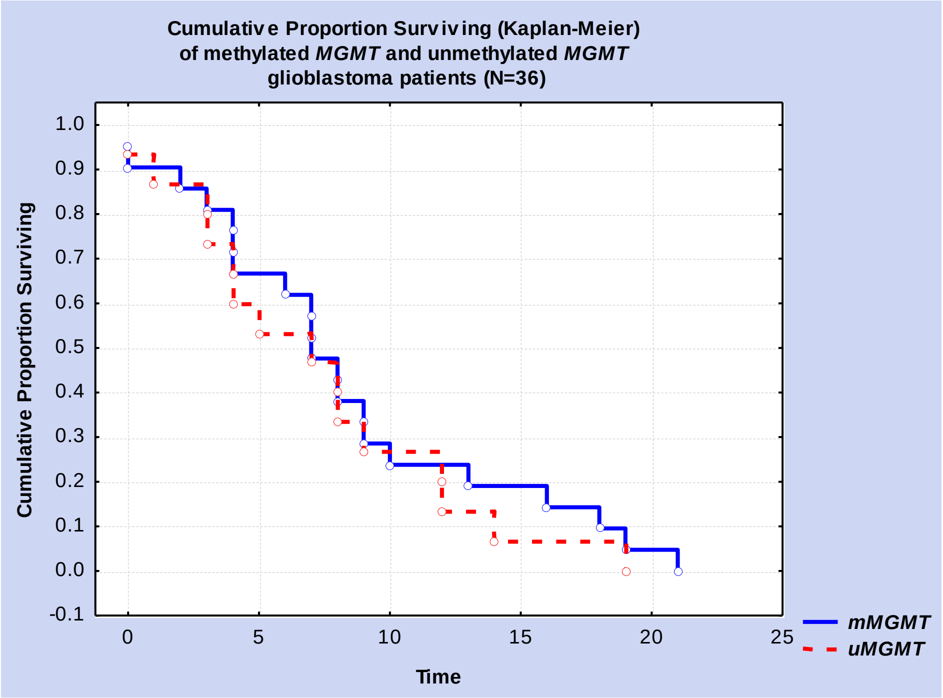

Figure S9. Kaplan-Meier curves of patients with glioblastoma (N=36)* – association of MGMT status estimated by combined MSP analysis and overall survival, considering the PMR=100% as cut-of value

*Long-term survival patient (80+ months) was excluded from analysis
